# In Vitro Hydrodynamic Assessment of a New Transcatheter Heart Valve Concept (the TRISKELE)

**DOI:** 10.1007/s12265-016-9722-0

**Published:** 2016-12-27

**Authors:** Benyamin Rahmani, Spyros Tzamtzis, Rose Sheridan, Michael J Mullen, John Yap, Alexander M. Seifalian, Gaetano Burriesci

**Affiliations:** 1 Cardiovascular Engineering Laboratory, UCL Mechanical Engineering, University College London, Torrington Place, London, WC1E 7JE UK; 2 Barts Health NHS Trust, University College London Hospital, London, UK; 3 NanoRegMed Ltd, London, UK; 4 Ri.MED Foundation, Bioengineering Group, Palermo, Italy

**Keywords:** TAVI, Self-expanding stent, Polymeric leaflets, Preclinical assessment

## Abstract

This study presents the in vitro hydrodynamic assessment of the TRISKELE, a new system suitable for transcatheter aortic valve implantation (TAVI), aiming to mitigate the procedural challenges experienced with current technologies. The TRISKELE valve comprises three polymeric leaflet and an adaptive sealing cuff, supported by a novel fully retrievable self-expanding nitinol wire frame. Valve prototypes were manufactured in three sizes of 23, 26, and 29 mm by automated dip-coating of a biostable polymer, and tested in a hydrodynamic bench setup in mock aortic roots of 21, 23, 25, and 27 mm annulus, and compared to two reference valves suitable for equivalent implantation ranges: Edwards SAPIEN XT and Medtronic CoreValve. The TRISKELE valves demonstrated a global hydrodynamic performance comparable or superior to the controls with significant reduction in paravalvular leakage. The TRISKELE valve exhibits enhanced anchoring and improved sealing. The valve is currently under preclinical investigation.

## Introduction

Transcatheter aortic valve implantation (TAVI) has evolved to become the standard treatment for inoperable and high-risk patients with severe aortic stenosis [1–3], accounting for more than 20% of global aortic valve replacement procedures [4, 5]. In intermediate-risk patients, TAVI has shown clinical outcomes and survival rates similar or superior to surgical aortic valve replacement [6–8]. However, further developments are still necessary to overcome technical and procedural challenges such as secure deployment and correct positioning of the prosthesis, common presence of paravalvular leakage [9, 10], frequent changes in atrioventricular conduction [11], difficulties in vascular access, embolization, and risk of stroke [12, 13]. More recently, there have been reports concerning possible subclinical leaflet thrombosis and reduced leaflet motion [14].

Chemically treated bovine and porcine pericardium are commonly used as leaflet material for TAVI valves, based on their broad and successful clinical history in surgical bioprostheses [15]. Nevertheless, the durability of bioprosthetic leaflets is still a matter of debate [16–18]. The nonphysiological stresses, applied to the leaflets of TAVI prostheses during crimping and deployment, cause tissue dehydration and are believed to increase the risk of structural damage [19–21], affecting adversely the durability of the pericardial valves [22–26]. Polymeric heart valves could represent an attractive alternative to xenograft tissues, addressing these limitations. Although no polymeric valve has reached the market yet, mainly due to their limited durability and hemocompatibility [27], recent advances in biomaterial science, surface modification techniques, prosthetic design, and fabrication methods have contributed to the development of new synthetic materials more suitable for valvular applications [28–30].

This study describes the in vitro hydrodynamic assessment of a new transcatheter heart valve concept, recently developed at UCL. The TRISKELE is a self-expanding valve with polymeric leaflets, aiming to mitigate complications related to imprecise valve positioning and provide a more reliable solution for both high risk patients with severe aortic stenosis and additionally the lower risk patient demographics at a lower cost.

## Methods

### Valve Description

**Fig. 1:**
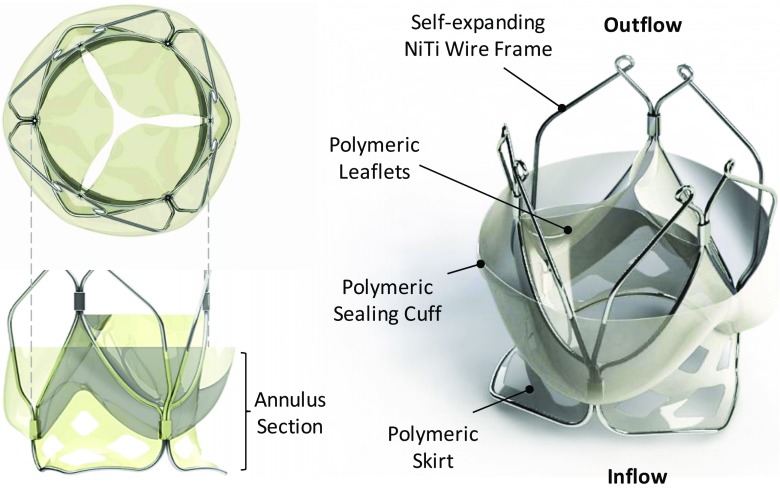
The TRISKELE transcatheter aortic valve

The TRISKELE valve is designed based on a self-expanding wire frame formed by thermomechanical shaping of three sets of nitinol wires mechanically joined by stainless steel crimping sleeves. The outflow portion of wire frame features three lateral ribs defined by sets of smoothly arched petal-like shapes that protrude radially further than the flow-control structure (Fig. 1). This geometrical feature helps maintain an open structure which reduces the impact on the surrounding tissue and dampens the pressure load transferred to the leaflets while functioning. The advantages of the TRISKELE delivery system has been previously described in detail [31]. The frame design was optimized numerically for three nominal sizes of 23, 26, and 29 mm, aiming at minimizing operating stresses and maintaining secure anchoring under conservative physiological pressure levels.

**Fig. 2:**
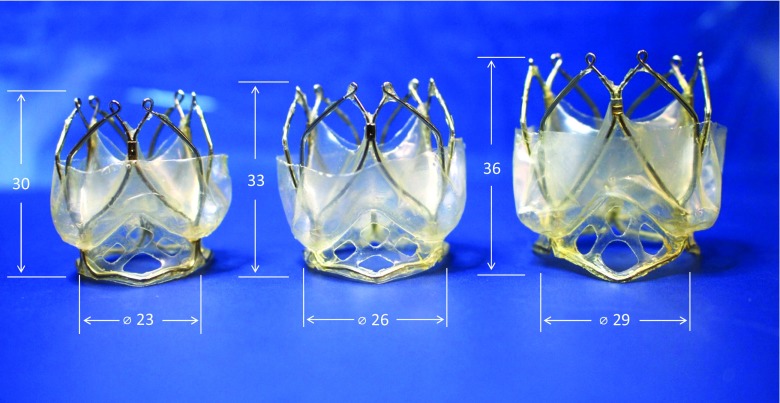
TRISKELE valve sizes 23 (*left*), 26 (*center*), and 29 (*right*). The automated manufacturing technique, developed in-house, facilitates consistent production of TRISKELE valves with a mean leaflet thickness of 130 ± 10 μm. The height and nominal diameter of TRISKELE valves are noted

The TRISKELE leaflets design is based on a novel principle successfully adopted previously for surgical tri-leaflets heart valves [32], which aims to achieve a single curvature in both the open and closed unloaded configurations. This approach has been shown to reduce the energy dissipated during the operating cycle, resulting in an improved hydrodynamic performance and reduced stress levels. Polyhedral oligomeric silsesquioxanes poly(carbonate-urea) urethane (POSS-PCU) was used to manufacture the leaflets and the sealing components of the TRISKELE valve. This is a nanocomposite polymer consisting of a hard crystalline segment and soft elastomeric segments in which polyhedral oligomeric silsesquioxanes (POSS) nanoparticles are attached as pendant chain functional groups to the backbone of poly(carbonate-urea) urethane (PCU). POSS-PCU has been previously validated in vitro for its hemocompatibility and anti-thrombogenicity [33–35], biostability [33], mechanical properties [36, 37], and resistance to calcification [38]. The leaflets and sealing cuff were manufactured using a new automated manufacturing technique developed in-house, which enables the construction of highly reproducible polymeric valves (Fig. 2) by robotic dip-coating of a stainless steel mandrel into an 18% (*w*/*v*) polymer solution. This manufacturing approach is suitable for a wide range of biostable polymers that can be used in combination or as an alternative to POSS-PCU.

The valve includes a skirt, supporting a flexible chalice shape sealing cuff departing from the skirt and surrounding the entire valve. The lower portion of the cuff is fixed with continuity to the valve skirt along a scalloped peripheral line, leaving the rest of the cuff free to adapt to the irregularity of the host interface (left ventricular outflow tract and native aortic leaflets) (Fig. 1) under the effect of the pressure difference between the aorta and the ventricle. The inflow portion of the skirt, below the sealing cuff, is fenestrated to reduce the stress in the polymer during crimping, and promote cell integration.

The geometrical dimensions of the TRISKELE valves of the currently available sizes are illustrated in Fig. 2.

### Hydrodynamic Assessment

**Fig. 3:**
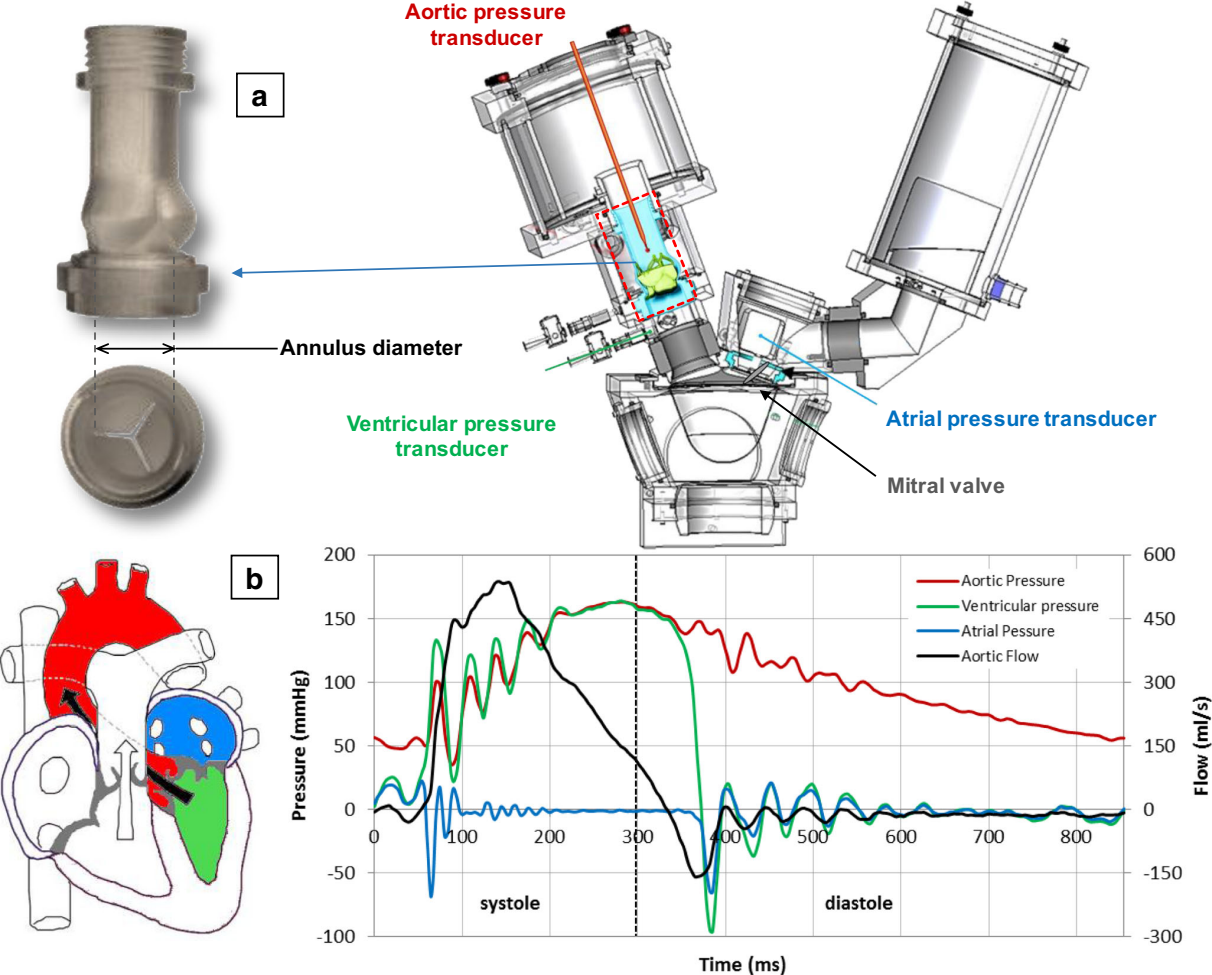
**a** The Vivitro pulse-duplicator system, consisting of a model left heart, a hydromechanical pump, flow measuring and data acquisition elements, and a mock aortic root designed to replicate the presence of the native valve; **b** a typical diagram of transvalvular pressures/flows over a cardiac cycle at CO of 5 l/min

In vitro bench tests were performed to evaluate the hydrodynamic function of the TRISKELE valves using a commercial cardiovascular pulse-duplicator (Vivitro Superpump System SP3891, Vivitro, Victoria, BC, Canada), reproducing physiologically equivalent aortic pressures and flows [39]. The pulse-duplicator was modified by incorporating a mock silicone aortic root based on the description provided by Swanson and Clark [40] inside the aortic chamber (Fig. 3a). The compliant aortic root compartments, developed in-house, include three cast rubber leaflets designed based on the dimensions and geometric relationships of the human aortic valve [41], replicating the presence of the native valve during the test. Mock aortic roots of 21-, 23-, 25-, and 27-mm annulus diameter were constructed to cover the recommended implantation ranges for all studied valve sizes. An additional bulged section was also included below the aortic annulus, to reproduce the ventricular outflow tract. In order to approximate calcific native tissues, common in TAVI applications, the selected root compliance for the testing pressure range was lower than 0.05% per mmHg, based on the definition in the ISO 5840 [39, 42].

Two commercially available TAVI valves, the Edwards SAPIEN XT (*n* = 1, per size) and the Medtronic CoreValve (*n* = 1, per size), were included in the study as controls, tested according to their manufacturer’s recommended implantation range guide.

All valves were tested in 37 °C buffered saline solution (0.90% *w*/*v* NaCl) at increasing cardiac output (COs) of 2, 3, 4, 5, 6, and 7 l/min, with a mean arterial pressure of 100 mmHg, a fixed heart rate of 70 beats per minute, and systole occupying 35% of the cardiac cycle [39]. Figure 3b shows a typical diagram of pressures and flows generated over a cardiac cycle at a CO of 5 l/min. Once the mean arterial pressure and the cardiac output flow readings stabilized, measurements of atrial, ventricular, and aortic pressures and aortic flow were collected and averaged over ten consecutive cardiac cycles. Based on these recordings, the mean transvalvular systolic pressure drop (∆P) and the regurgitant fraction were determined. The regurgitant fraction represents the total regurgitant volume expressed as a percentage of the stroke volume. The total regurgitant volume is the sum of the *closing regurgitant* volume, associated with the dynamic of valve closure, and the *leakage regurgitant* volume, corresponding to the leakage through the closed valve (in the case of TAVI devices, it can be essentially attributed to paravalvular leakage). Effective orifice area (EOA), which represents the minimal cross-sectional area of the downstream jet at the aortic valve orifice [43], was derived from the continuity equation, applying Gorlin’s formula [44]. The fluid-mechanical left ventricular energy loss associated with the valve was calculated as the time integral of the product of the mean systolic pressure drop and aortic flow, over the different phases of the cardiac cycle [39]. The energy loss value quantifies the overall impact of the valve performance on the myocardial function [45], taking into account the losses associated with both pressure drop during systole and the regurgitation during diastole. Data are presented in mean ± standard deviation (*n* = 3, per size of the TRISKELE valves).

## Results

### Aortic Root Size 21

**Fig. 4:**
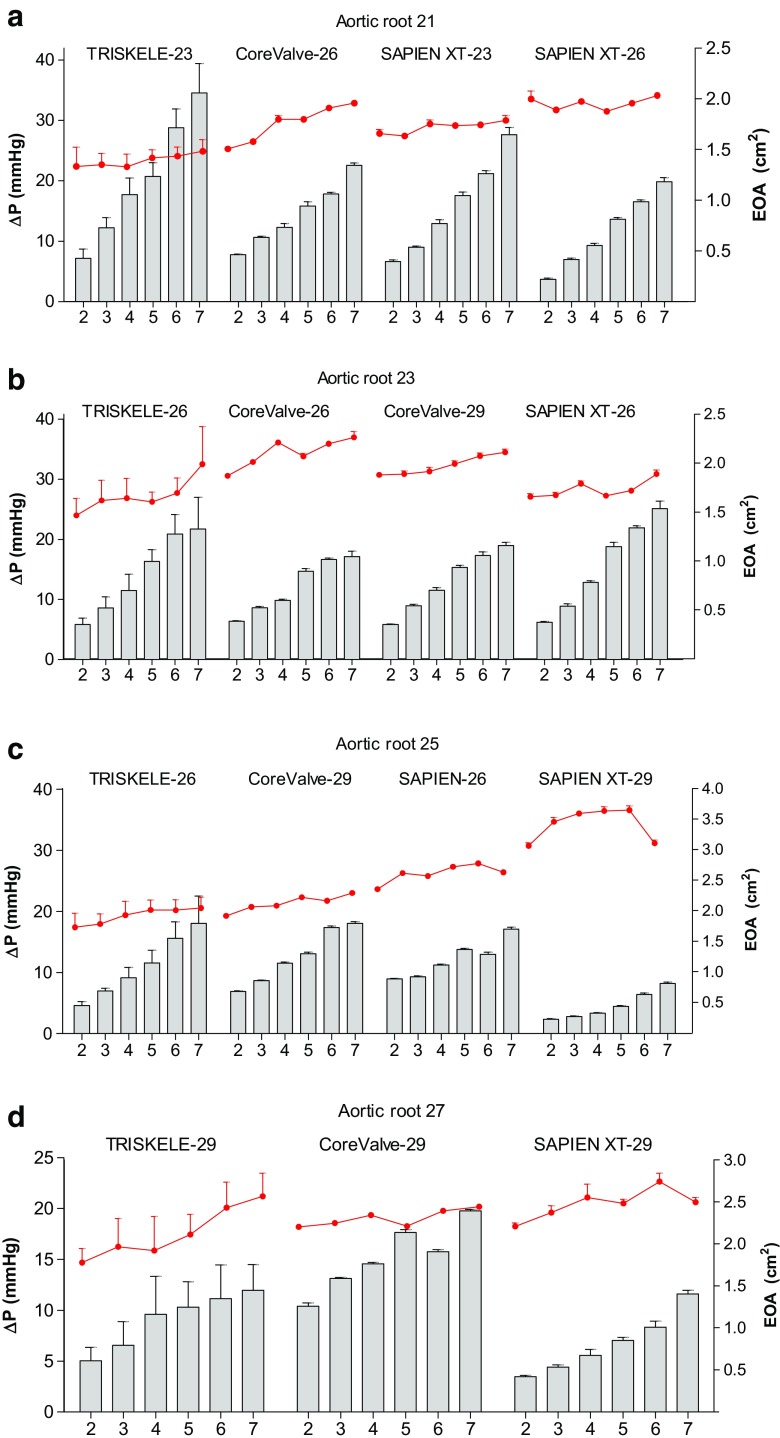
Transvalvular pressure drop (ΔP) (*bars*, left *Y*-axis units) and effective orifice area (points and connecting lines, right *Y*-axis units) measured as a function of cardiac output (2–7 l/min) in mock aortic roots of 21 mm (**a**), 23 mm (**b**), 25 mm (**c**), and 27 mm (**d**) annulus. Data are expressed as mean ± SD, *n* = 30 for TRISKELE (three valves, each tested over 10 cycles) and *n* = 10 for control valves (one valve tested over 10 cycles)

**Fig. 5:**
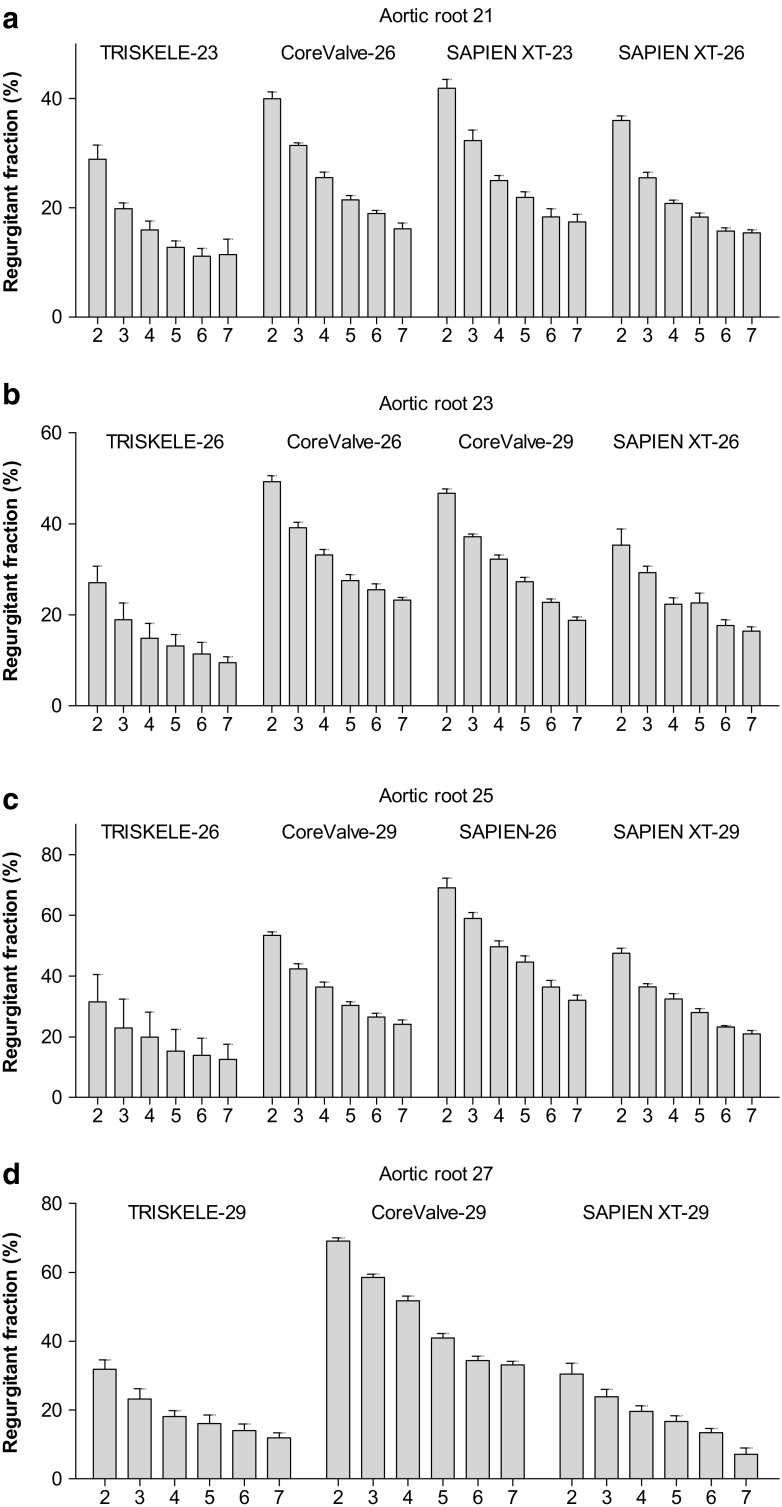
Total regurgitant fraction measured as a function of cardiac output (2–7 l/min) in mock aortic roots of 21 mm (**a**), 23 mm (**b**), 25 mm (**c**), and 27 mm (**d**) annulus. Data are expressed as mean ± SD, *n* = 30 for TRISKELE (three valves, each tested over 10 cycles) and *n* = 10 for control valves (one valve tested over 10 cycles)

**Fig. 6:**
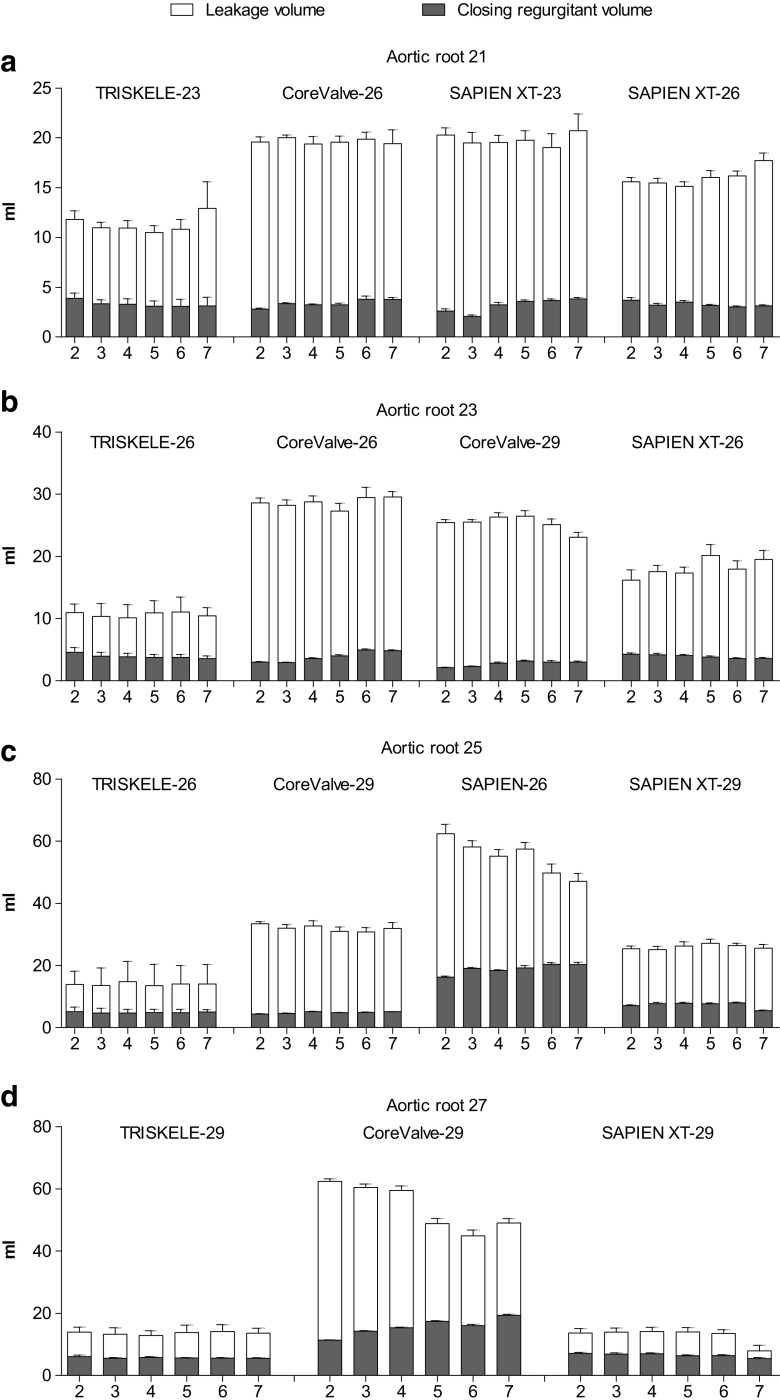
Components of the regurgitant volume measured as a function of cardiac output (2–7 l/min) in mock aortic roots of 21 mm (**a**), 23 mm (**b**), 25 mm (**c**), and 27 mm (**d**) annulus. Data are expressed as mean ± SD, *n* = 30 for TRISKELE (three valves, each tested over 10 cycles) and *n* = 10 for control valves (one valve tested over 10 cycles)

**Fig. 7:**
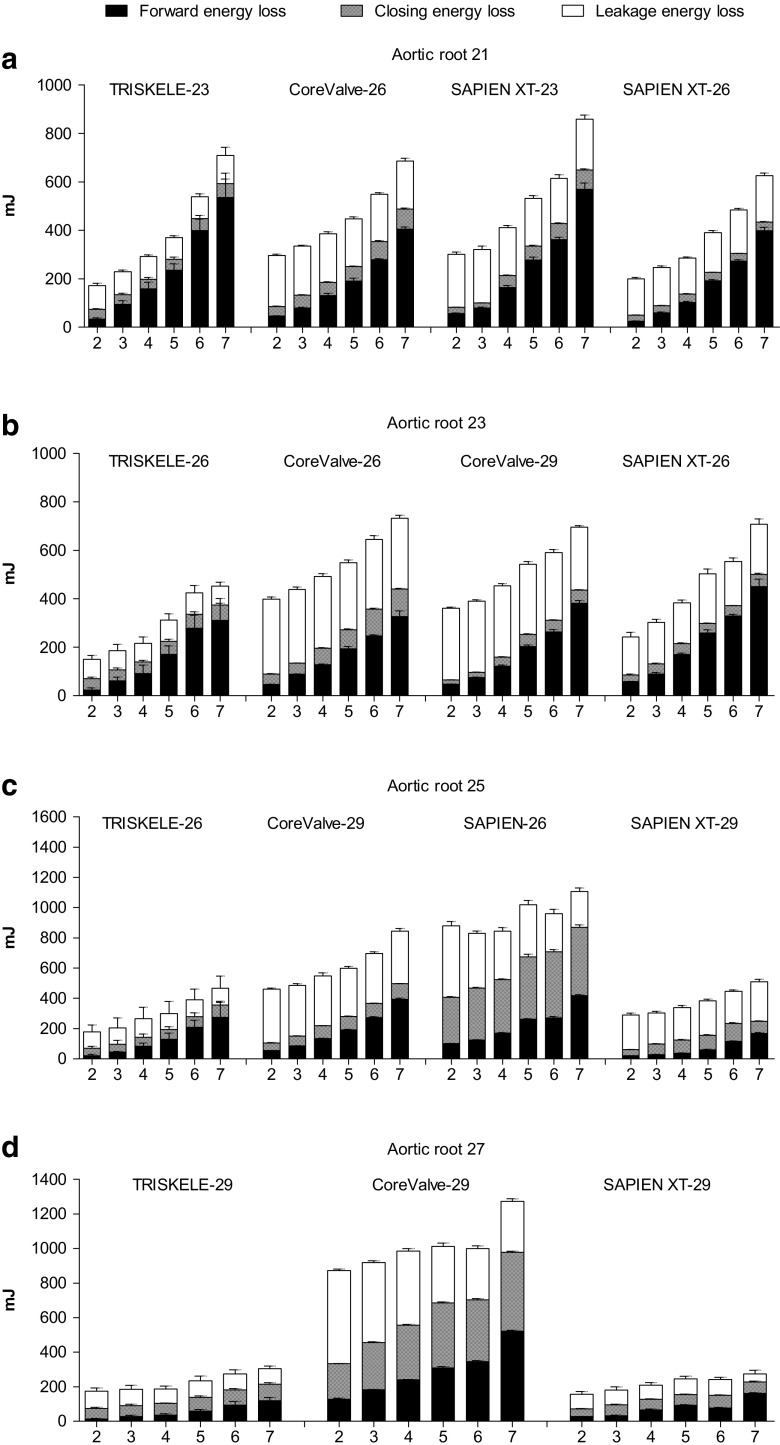
Fluid-mechanical left ventricular energy loss, calculated at increasing cardiac output (2–7 l/min) in mock aortic roots of 21 mm (**a**), 23 mm (**b**), 25 mm (**c**), and 27 mm (**d**) annulus. Data are expressed as mean ± SD, *n* = 30 for TRISKELE (three valves, each tested over 10 cycles) and *n* = 10 for control valves (one valve tested over 10 cycles)

In the 21-mm aortic root, the size 23 TRISKELE valves were slightly more constricted than the controls, resulting in a relatively higher transvalvular pressure drop (Fig. 4a). At lower COs, the TRISKELE-23 had a similar ∆P to those of the control valves but showed higher relative pressure gradient as the CO increased. The mean ∆P over the entire CO range was measured as 20.2, 14.5, 15.8, and 11.7 mmHg, while the mean EOA was 1.4, 1.8, 1.7, and 2.0 for the TRISKELE-23, CoreValve-26, SAPIEN-23, and SAPIEN-26, respectively. The mean regurgitant fraction was 16.7, 25.6, 26.1, and 21.9% (Fig. 5a), with similar closing volumes, but leakage volumes of 8.0, 16.3, 16.6, and 12.7 ml measured for the TRISKELE-23, CoreValve-26, SAPIEN-23, and SAPIEN-26, respectively (Fig. 6a). The TRISKELE-23 demonstrated a minimal total energy loss of 384 mJ, similar to SAPIEN-26 (372 mJ), and significantly better than CoreValve-26 (450 mJ) and SAPIEN-23 (506 mJ) (Fig. 7a).

### Aortic Root Size 23

In the 23-mm aortic root, the mean ∆P over the entire COs measured as 14.1, 12.2, 13, and 15.6 mmHg, with mean EOAs of 1.7, 2.1, 2.0, and 1.7 cm^2^ achieved by the TRISKELE-26, CoreValve-26, CoreValve-29, and SAPIEN-26, respectively (Fig. 4b). Frequent ventricular migration was observed for the CoreValve at CO higher than 3 l/min. Therefore, it was constrained to the distal end of the aortic root using downstream tethers to prevent premature migration to ventricular chamber. The TRISKELE-26, CoreValve-26, CoreValve-29, and SAPIEN-26 were performed with mean regurgitant fractions of 15.8, 33.0, 30.8, and 23.9% (Fig. 5b); similar closing volumes; mean leakage volumes of 6.7, 24.7, 22.6, and 14.2 ml (Fig. 6b); and mean total energy losses of 278, 543, 506, and 449 mJ, respectively (Fig. 7b).

### Aortic Root Size 25

In the 25-mm aortic root, the TRISKELE-26, CoreValve-29, SAPIEN-26, and SAPIEN-29 were associated with mean ∆P over the entire COs of 11.0, 12.6, 12.2, and 4.6 mmHg, and mean EOAs of 1.9, 1.1, 2.6, and 3.4 cm^2^, respectively (Fig. 4c). Valve migration was observed for both the CoreValve and SAPIEN-26 (migrated at a Co of 6 l/min). Hence, both valves were constrained to the aortic root. The mean regurgitant fraction was measured as 19.3, 35.5, 48.4, and 31.4% (Fig. 5c), with 4.9, 4.8, 18.9, and 7.3 ml of closing regurgitant volume, and 9.1, 27.2, 36.1, and 18.7 ml of leakage volumes (Fig. 6c)for the TRISKELE-26, CoreValve-29, SAPIEN-26, and SAPIEN-29, respectively. In the same order, these valves experiences mean total energy losses of 301, 606, 940, and 379 mJ (Fig. 7c).

### Aortic Root Size 27

In the 27-mm aortic root, the mean ∆P over the entire COs measured as 9.1, 15.2, and 6.7 mmHg, with mean EOAs of 2.1, 2.3, and 2.5 cm^2^ recorded for the TRISKELE-29, CoreValve-29, and SAPIEN-29, respectively (Fig. 4d). In the same order, these valves were associated with regurgitant fraction of 19.2, 48.0, and 18.5% (Fig. 5d); closing regurgitant volumes of 5.7, 15.6, and 6.5 ml; leakage volumes of 7.9, 38.6, and 6.4 ml (Fig. 6d); and total energy losses of 227, 1010, and 218 mJ (Fig. 7d). The CoreValve-29 was constrained in this aortic root as well.

## Discussion

This study provided important data on the function and flow characteristics of the TRISKELE valves, as well as the reference devices. The valves were implanted in the mock aortic roots following their intended loading and deployment steps (the reference valves were implanted in accordance with their manufacturer’s instructions for use), and tested in pulsatile setup simulating a wide range of flow conditions over increasing cardiac output of 2–7 l/min. The self-expanding valves, the TRISKELE and the CoreValve, experienced some flow-induced self-readjustment from their initial implantation position while reaching physiologic pressure and flow conditions.

In general, the TRISKELE valves had comparable performance with the control valves during systole, with exception for the 21-mm annulus aortic root, where our study device exhibited appreciably higher levels of ∆P and worse EOA than the controls (Fig. 4a). This can be attributed to the adopted anchoring approach, designed to reduce the radial forces while enhancing anchoring, which may produce some overconstraint of the leaflets commissures at the smallest implantation sizes, compared to the reference valves. Nevertheless, this limitation could be corrected by expanding the available sizes for the valve to lower diameters.

The SAPIEN valves demonstrated comparatively better performance in small implantation sizes (21 and 23 mm), probably due to the fact that this prosthesis, being balloon expandable, has a stiffer stent which retains a larger EOA after inflation of the balloon and successive partial recoil. TAVI prostheses typically operate at a configuration that is smaller than their fully expanded forms. Once implanted, the functional orifice area of a these valves may be limited by the stenotic native valve leaflets and/or the inner diameter of the diseased bioprostheses (in the case of valve-in-valve procedure) [46–48]. The self-expanding stents may be associated with a relatively smaller EOA, as they are often deformed under the radial anchoring forces [49].

Common recurrence of paravalvular regurgitation is a major drawback of current TAVI valves [9, 10, 50, 51]. The TRISKELE demonstrated superior performance during diastole, achieving major reduction in paravalvular leakage in all aortic roots. The sealing cuff incorporated into the TRISKELE design minimizes the paravalvular leakage by covering the gaps between the prosthetic and native tissue. Almost all sizes of the TRISKELE valves achieved a significantly lower total regurgitation (closing regurgitation and leaking volume) compared to the control valves.

The systolic hydrodynamic function of TAVI valves may be as good as or even exceed the performance of surgically implanted bioprostheses [52]; however, their diastolic performance is often compromised by paravalvular leakage and transvalvular regurgitation, resulting in increased energy loss during diastole [53]. Energy loss measurements provide a comprehensive criteria to evaluate and compare the overall hydrodynamic performance of the valves during an entire cardiac cycle, based on their effect on the ventricular workload [45]. Higher paravalvular leakage and central regurgitation have an adverse impact upon valve performance. The lower total energy loss achieved by the TRISKELE indicates a lesser fraction of the ventricular workload to operate, when compared to the reference valves.

Both reference valves experienced some form of dislodgement during the tests, which compromised their performance within the recommended implantation ranges, with the CoreValves having more frequent migrations at CO higher than 3 l/min. In these cases, it was only possible to determine the hydrodynamic performance of the functional components by “artificially” constraining the valves in their initial axial position, although this was not ideal. These premature migrations may be, to some extent, associated with the absence of calcific or fibrotic lesions in the in vitro test model and the smooth surface of the silicone leaflets in the mock aortic roots. Similar observations have been reported previously in animal studies [54, 55].

No migration was observed for the TRISKELE valves, which contrary to the reference devices is secured to the implantation site by applying mainly counteracting axial forces, rather than radial actions [49]. The lower protrusions of the frame (inflow portion) project into the left ventricle and the upper petal-like ribs (outflow portion) expand radially on top of the valve annulus, into the leaflets of the native valve (Fig. 1). The valve anchoring is achieved by constraining the aortic annulus and not by dilating it, thus avoiding the application of high levels of distributed radial forces which might perturb the atrioventricular node and the left bundle branch. This approach is suitable for application in patients with calcific stenosis, as well as for uncalcified anatomies, as confirmed by the successful implants in healthy and compliant native ovine aortic valve [31].

In an attempt to replicate the presence of stiffened native cusps, the mock aortic roots used in this study were made with the same thickness as the root wall. This is, necessarily, an approximation, as it does not incorporate the surface irregularities produced by the presence of calcific nodules, which possibly contribute, in vivo, to a more effective retention of the reference valves. However, the model provides an idealized configuration, which allows direct comparison of different valves under identical anatomical and operating conditions.

Polymeric leaflets provide more design freedom compared to industry standard xenografts. Biostable polymers can be used to produce leaflets two to three times thinner than pericardial tissues, which can potentially result in smaller collapsible valve profile and minimize complications related to vascular access. The thinner polymeric leaflets also improve the hemodynamics of the valve, making it easier to open and achieve a larger orifice area [56].

Biostable polymers can be engineered to exhibit a tailored range of physical, chemical, and biological characteristics. The α-Gal-free nature of biostable polymers means lower risks of calcification compared to porcine and bovine tissues and may lead to potential advantages in terms of long-term performance and durability [38, 57, 58]. Moreover, there is no suturing involved in the manufacturing process, hence no stitch hole in the leaflets which could cause tissue tear in the flexion zones. Pericardial tissues are reported to be prone to dehydration and collagen fiber impairment [22–26]. Polymeric leaflets are less susceptible to physical damage induced as a result of collapsing/crimping, hence can be preloaded in air long before the operation, without causing any observable harm to the valve [31].

While new TAVI devices with improved delivery strategies are being developed, there are both hopes and concerns on expanding the application of TAVI to less risky patient demographics [6, 59]. The future of transcatheter heart valve replacement and its expansion to lower risk patients depends on technological advances in biomaterials, anticalcification treatment, and development of collapsible valves for optimal delivery and deployment.

## Limitations

Isotonic saline was used as testing fluid in this study, which is less viscous than blood, and results into increased leakage volumes compared with the clinical cases. Although fluid viscosity is reported to have little influence on systolic performance of the test valves, it may affect the leakage flow in an in vitro setup [60, 61]. Another limitation of this study was the lack of statistical power due to minimal number of reference devices (i.e., SAPIEN XT and CoreValve). A larger sample size is required to ensure adequate representation of the expected variability in the control group.

Also, it is worth to mention that significant efforts have been made by TAVI manufacturers to address the recurrences of paravalvular leakage, and a number of latest generation transcatheter valves, including the newest member of the Edwards SAPIEN family (SAPIEN 3), are reported to have reduced paravalvular leakage compared to the reference valves used in this study [62].

## Conclusion

An in vitro study was performed to evaluate the hydrodynamic function of a new transcatheter heart valve concept, with polymeric leaflets, an adaptive sealing cuff, and a novel fully retrievable self-expanding frame. The TRISKELE prototypes of 23, 26, and 29 nominal diameter were manufactured using a new automated technique which allows manufacturing of highly reproducible polymeric valves. The TRISKELE valves exhibited comparable or superior hydrodynamic performance compared to the SAPIEN XT (Edwards Lifesciences) and the CoreValve (Medtronic) devices, when tested in appropriate annuli (21-, 23-, 25-, and 27-mm diameter). The TRISKELE valve demonstrated significant reduction in paravalvular leakage (independently on the presence of calcification). The satisfactory results from this study encourage the possibility for further development and refinement. The TRISKELE valve is currently under preclinical investigation for its durability and function in chronic animal studies.

## Abbreviations

ΔP: Transvalvular systolic pressure drop
CO: Cardiac output
EOA: Effective orifice area
TAVI: Transcatheter aortic valve implantation
POSS: Polyhedral oligomeric silsesquioxanes
PCU: Poly(carbonate-urea) urethane
UCL: University College London
